# Serologically-Based Evaluation of Cross-Protection Antibody Responses among Different A(H1N1) Influenza Strains

**DOI:** 10.3390/vaccines8040656

**Published:** 2020-11-05

**Authors:** Serena Marchi, Ilaria Manini, Otfried Kistner, Pietro Piu, Edmond J. Remarque, Alessandro Manenti, Fabrizio Biuso, Tommaso Carli, Giacomo Lazzeri, Emanuele Montomoli, Claudia Maria Trombetta

**Affiliations:** 1 Department of Molecular and Developmental Medicine, University of Siena, 53100 Siena, Italy; serena.marchi2@unisi.it (S.M.); ilaria.manini@unisi.it (I.M.); giacomo.lazzeri@unisi.it (G.L.); emanuele.montomoli@unisi.it (E.M.); 2 Interuniversity Research Center on Influenza and Other Transmissible Infections (CIRI-IT), 16132 Genoa, Italy; 3 VisMederi srl, 53100 Siena, Italy; kistner@vismederi.com (O.K.); pietro.piu@vismederi.com (P.P.); alessandro.manenti@vismederiresearch.com (A.M.); biuslee@yahoo.it (F.B.); tommaso_carli@yahoo.com (T.C.); 4 Department of Virology, Biomedical Primate Research Centre, 2288 Rijswijk, The Netherlands; remarque@bprc.nl; 5 VisMederi Research srl, 53100 Siena, Italy

**Keywords:** influenza virus, H1N1, immunity, antigenic drift

## Abstract

After the influenza H1N1 pandemic of 2009, the seasonal A/Brisbane/59/2007 strain was replaced by the A/California/07/2009 strain for the influenza virus vaccine composition. After several seasons with no indications on the occurrence of antigenic drift, A/Michigan/45/2015 was chosen as the H1N1 vaccine strain for the 2017/2018 season. Since the immune response to influenza is shaped by the history of exposure to antigenically similar strains, the potential cross-protection between seasonal human influenza vaccine strains and the emerging pandemic strains was investigated. Human serum samples were tested by hemagglutination inhibition and single radial hemolysis assays against A/Brisbane/59/2007, A/California/07/2009, and A/Michigan/45/2015 strains. Strong cross-reactions between A/California/07/2009 and A/Michigan/45/2015 strains were observed in 2009/2010, most likely induced by the start of the 2009 pandemic, and the subsequent post-pandemic seasons from 2010/2011 onward when A/California/07/2009 became the predominant strain. In the 2014/2015 season, population immunity against A/California/07/2009 and A/Michigan/45/2015 strains increased again, associated with strong cross-reactions. Whereas hemagglutination inhibition assay has a higher sensitivity for detection of new seasonal drift, the single radial hemolysis assay is an excellent tool for determining the presence of pre-existing immunity, allowing a potential prediction on the booster potential of influenza vaccines against newly emerging drifted strains.

## 1. Introduction

The immune response to influenza is shaped by the individual history of exposure to related antigens. Individuals who have been exposed to a greater number of influenza strains, through natural infection or vaccination, likely harbor pre-existing memory responses that cross-react with vaccine strains.

A new A/H1N1 influenza virus began circulating in humans in the spring of 2009, causing the first pandemic of the 21st century. The new virus (A/California/07/2009 H1N1pdm09) was the result of a triple reassortment from human, swine, and Eurasian avian influenza viruses and affected mostly children and young adults [1,2]. This could be due to the similarity of the H1N1 pdm09 strain to the viruses circulating in humans between the 1930s and 1950s, suggesting that adults, in particular those over 60 years old, could have some cross-reactive antibody responses against the pandemic virus [3,4,5,6], as proven by several studies [3,4,7,8,9,10,11].

The recommendation for the H1N1 component for the influenza vaccines changed from A/New Caledonia/20/1999-like strain in seasons 2005/2006 and 2006/2007 to A/Solomon Island/03/2005-like strain in season 2007/2008 and subsequently to A/Brisbane/59/2007-like strain in the 2008/2009 and 2009/2010 seasons. However, in 2009, A/California/07/2009 emerged as a pandemic strain and replaced A/Brisbane/59/2007 as the H1N1 circulating strain. Consequently, the World Health Organization (WHO) provided a vaccine recommendation for A/California/07/2009 as the seasonal H1N1 vaccine strain after the end of the pandemic. In 2017/2018 season, the WHO recommendation changed to A/Michigan/45/2015-like (Table 1) [12]. Here, we refer to the recommendations until 2009/2010 as “pre-pandemic,” 2009 and 2010 as “pandemic,” and from season 2010/2011 onward as “post-pandemic.”

Hemagglutinin (HA) and neuraminidase (NA) are the two major surface glycoproteins of influenza viruses, both recognizing sialic acid (SA). HA binds to SA on the host cells, allowing virus entry, whereas NA has enzymatic activity removing SA, facilitating the release of progeny virus [13]. HA is the major target of humoral immune response and rapid antigenic variation due to the accumulation of mutations results in antigenic drift. In addition, periodic reassortment between antigenically distinct influenza viruses can lead to antigenic shift and the emergence of pandemic strains [14]. The HA molecule comprises a membrane-distal domain (globular head, HA1) and a membrane-proximal domain (stalk, HA2). Most antibody responses against HA are strain-specific, targeting HA1. Alternatively, HA2 is highly conserved compared with the globular head, making it a suitable target for vaccine development to induce broad and protective immune responses [15].

The immunological response to influenza vaccine and natural infection is mainly evaluated by two serological techniques, hemagglutination inhibition (HI) and single radial hemolysis (SRH), both of which are still the most widely used and officially recognized serological assays by regulatory authorities. In recent years, the importance of neutralization assays for serological evaluations has increased and the analysis of neuraminidase-specific antibodies has also become a topic of increasing interest [16]. However, the HI assay is still considered the gold standard for antigenic and serological analysis [17]; it still widely serves as a correlate of protection for influenza vaccines in detecting antibodies able to inhibit the interaction between red blood cells (RBCs) and the globular head of HA, i.e., blocking of receptor-binding activities and subsequent inhibition of infection events. The detected antibodies seem to be strain-specific and not protective against mismatching influenza strains [16].

Alternatively, the SRH assay measures complement fixation antibodies, mainly immunoglobulin (Ig) G (IgG1 and IgG3), not only against the surface glycoproteins HA and NA, but also against internal antigens, leading to a potential lack of specificity to HA antibodies [18,19].

For decades, an HI titer ≥40 and a SRH hemolysis area of 25 mm^2^ or greater have been acknowledged as correlates of protection and were considered the protective threshold level beyond which the probability of contracting influenza infection was reduced by 50% or more. Since February 2017, the revised guidelines on influenza vaccines by the European Medicine Agency (EMA) have withdrawn the traditional concept of HI and SRH as correlates of protection [20], in contrast to other representative regulatory authorities such as United States Food and Drug Administration (U.S. FDA). However, the debate on HI and/or SRH representing a correlate of protection (CoP) or at least a surrogate of protection (SoP) is still ongoing [16].

The aim of this study was to investigate the specificity of the HI and SRH assays with respect to influenza virus antigenic drift and shift variants and potential cross-reactivity between seasonal human influenza H1N1 vaccine strains and the emerging pandemic H1N1 strain of swine origin. Therefore, A/Brisbane/59/2007 was chosen as the last recommended H1N1 strain of the pre-pandemic seasons until 2008/2009, representing the H1N1 viruses circulating in the human population since then. The pandemic strain A/California/07/2009 was chosen as the representative strain for the 2009/2010 pandemic season and the post-pandemic seasons from 2010/2011 onward, including A/Michigan/45/2015 as a drift variant of A/California/07/2009, recommended by the WHO as the H1N1 vaccine strain of the 2017/2018 season.

## 2. Materials and Methods

### 2.1. Virus Antigens

The infectious influenza A/H1N1 viruses used for serological assays were seasonal influenza strains obtained from the National Institute for Biological Standards and Control (NIBSC): A/Brisbane/59/2007 (H1N1, 09/276), A/Michigan/45/2015 (H1N1, 16/354), and A/California/07/2009 (H1N1, 09/216). All viruses used were egg-grown.

### 2.2. Serum Samples

Human serum samples were obtained from the Sera Bank of the Laboratory of Molecular Epidemiology, Department of Molecular and Developmental Medicine, University of Siena, Siena, Italy. The samples were anonymously collected and stored in compliance with Italian ethics law.

Serum samples were collected from the general population in Italy between January 2005 and August 2017. Out of 1000 samples available for every single season, 100 samples were randomly selected for each season included in this study (2005/2006, 2008/2009, 2009/2010, 2010/2011, 2013/2014, 2014/2015, 2015/2016, and 2016/2017) balanced between two age groups, 18–65 years old (younger adults, *n* = 50) and >65 years old (elderly adults, *n* = 50).

### 2.3. Hemagglutination Inhibition Assay

All serum samples were pre-treated with receptor destroying enzyme (RDE) (ratio 1:5) from Vibrio Cholerae (Sigma Aldrich, St. Louis, MO, USA) for 18 h at 37 °C in a water bath and then heat inactivated for 1 h at 56 °C in a water bath with 8% sodium citrate (ratio 1:4).

Turkey RBCs (TRBCs) were centrifuged two times, washed with 0.9% saline solution, and adjusted to a final dilution of 0.35%.

From an initial dilution of 1:10, serum samples were 2-fold diluted in duplicate with 0.9% saline solution in a 96-well plate. Twenty-five microliters of standardized viral antigen were added to each well and the mixture was incubated at room temperature for one hour. TRBCs were added and, after one hour of incubation at room temperature, the plates were evaluated for the presence of agglutination inhibition.

The antibody titer is expressed as the reciprocal of the highest serum dilution showing complete inhibition of agglutination. Since the starting dilution was 1:10, the lower limit of detection (LoD) for the antibody titer is 10. When the titer was under the detectable threshold, the results were conventionally expressed as 5 for calculation purposes (half the lowest detection threshold).

### 2.4. Single Radial Hemolysis Assay

Serum samples were heat inactivated at 56 °C for 30 min in a water bath.

Fresh TRBCs were centrifuged and washed with phosphate buffer saline (PBS) twice. Diluted virus antigen was added to the TRBCs suspension at a concentration of 2000 hemagglutinin units (HAU) per mL. To allow the adsorption of viral antigen to the TRBCs, the suspension was incubated at 4 °C for 20 min. A solution of 2.5 mM chromium chloride (CrCl_3_) was added to the previous suspension and incubated at room temperature for 10 min to increase the binding affinity between the TRBCs and the viral antigen. The suspension was carefully mixed and centrifuged. The supernatant was removed and PBS was added to the pellet, which was then carefully re-suspended. A stock solution of 1.5% agarose in PBS containing 0.1% sodium azide and 0.5% low gelling agarose was prepared. The agarose stock solution was kept at 45 °C in a water bath.

Each SRH plate consisted of TRBCs—viral antigen suspension and guinea pig complement in the agarose mixture. The final suspension was homogenized by shaking and spread onto each plate, incubated at room temperature for 30 min and then stored at 4 °C for 30–90 min to set the agarose. Holes were punctured in each plate with a calibrated punch and 6 µL of serum samples and controls added into each hole. The plates were stored in a humid box and incubated at 4 °C for 16–18 h in the dark. After overnight incubation, the plates were further incubated in a water bath at 37 °C for 90 min and then the diameters of hemolysis areas read in millimeters with a calibrating viewer [19].

### 2.5. Statistical Analysis

All statistical analyses were performed using Microsoft R-Open version 3.5.0 (R Core Team, 2018, city, country). R is a language and environment for statistical computing (R Foundation for Statistical Computing, Vienna, Austria). For the purpose of this study, only samples with an HI titer ≥ 40 and SRH hemolysis area ≥ 25 mm^2^ were considered positive.

The results from the HI and SRH assays are reported as a proportion of positive samples along different seasons’ antigens, all ages, and age groups (18–65 years old and >65 years old) separately. Corresponding 95% confidence intervals (CIs) were calculated by the Wilson method using the DescTools R-package. Venn diagrams reporting proportions of cross-protection were prepared using the VennDiagram R-package. A chi-square test was used to compare proportions of positives. Statistical significance was set at *p* < 0.05, two-tailed.

### 2.6. Influenza Hemagglutinin Multiple Sequence Alignments

Multiple sequence alignments were performed using the Basic Local Alignment Search Tool (BLAST) server [21]. HA sequences of A/Brisbane/59/2007 (GenBank accession: CY030234) and A/Michigan/45/2015 (Genbank accession: MK622940) were compared against the HA sequence of A/California/07/2009 (Genbank accession: GQ280797). The similarity between sequences is expressed as a percentage.

## 3. Results

**Figure 1:**
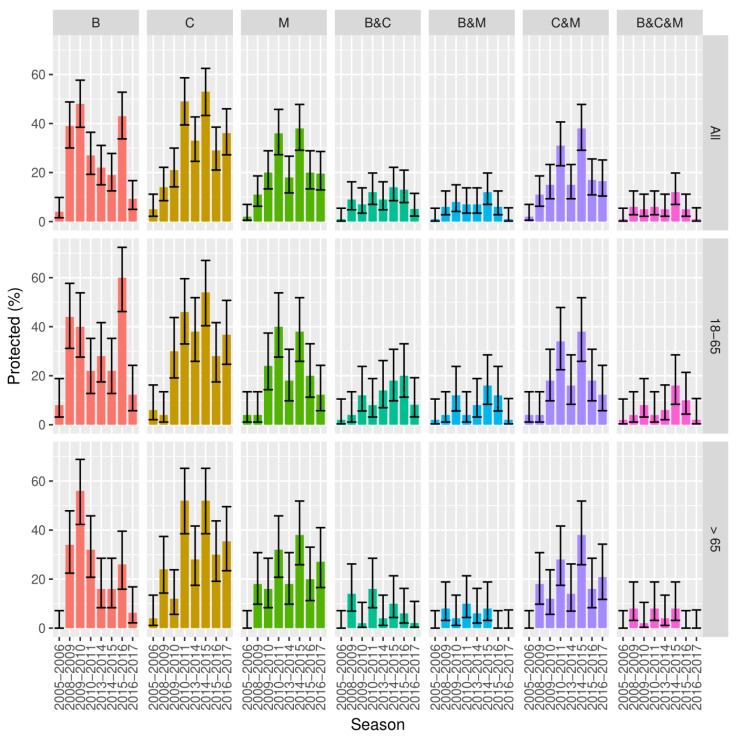
HI proportions of subjects with protective titers with 95% CI by strain (A/Brisbane, B; A/California, C; A/Michigan, M) and combinations by age group and season.

**Figure 2:**
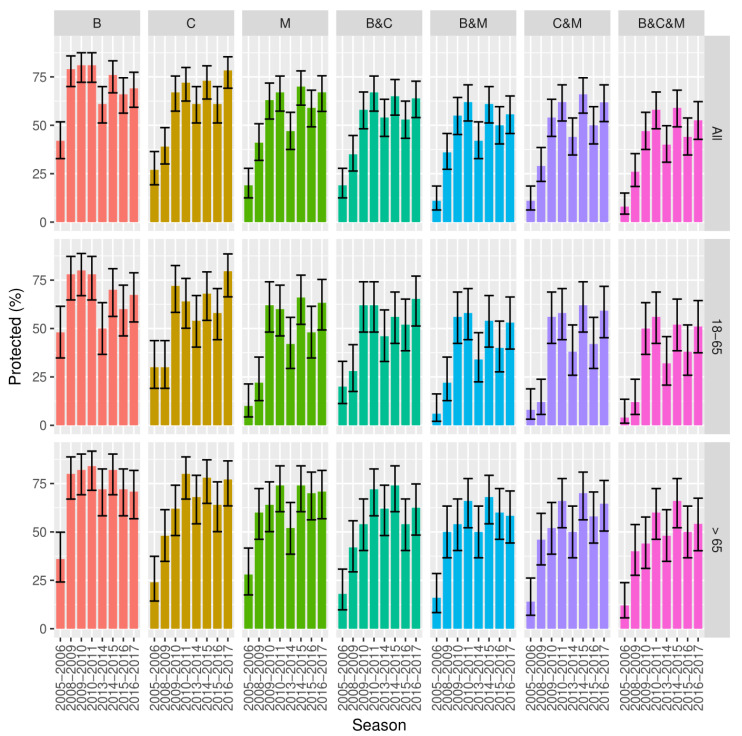
SRH proportions of subjects with protective titers with 95% CI by strain (A/Brisbane, B; A/California, C; A/Michigan, M) and combinations by age group and season.

The results by HI and SRH assays against human influenza H1N1 vaccine strains A/Brisbane/59/2007 (referred to as A/Brisbane), A/California/07/2009 (referred to as A/California), and A/Michigan/45/2015 (referred to as A/Michigan) are shown in Figure 1 and Figure 2.

Between the 2005/2006 and 2008/2009 seasons, the A/Brisbane strain showed a significant increase in the proportion of positive subjects (39.0%, 95% CI 30.0–49.0 for HI and 79.0%, 95% CI 70.0–86.0 for SRH; *p* < 0.0001 for both assays). In the following years, the proportion of HI-positive subjects against the A/Brisbane strain decreased, with the exception of a peak observed for the 2015/2016 season (43.0%, 95% CI 34.0–53.0; *p* = 0.0002 vs. 2014/2015 for HI results). In contrast, variations in the proportion of positive subjects were lower in SRH.

The A/California strain showed a trend characterized by two different peaks: the first in 2010/2011 (49.0%, 95% CI 39.0–59.0; *p* < 0.0001 vs. 2009/2010 for HI results) and the second one in 2014/2015 (53.0%, 95% CI 43.0–62.0; *p* = 0.004 vs. 2013/2014 for HI results). As observed for the A/California strain, the A/Michigan strain also showed a peak in the 2010/2011 (36.0%, 95% CI 27.0–46.0; *p* = 0.012 vs. 2009/2010 for HI results) and 2014/2015 (38.0%, 95% CI 29.0–48.0; *p* = 0.002 vs. 2013/2014 for HI results) seasons. Considering the SRH results, both strains showed a significant increase in the 2009/2010 season (67.0%, 95% CI 57.0–75.0 for A/California and 63.0%, 95% CI 53.0–72.0 for A/Michigan; *p* < 0.0001 and *p* = 0.002 vs. 2008/2009, respectively), whereas for 2014/2015, only an increase in A/Michigan was observed (70.0%, 95% CI 60.0–78.0; *p* = 0.002 vs. 2013/2014). In general, the proportions of positive subjects for A/Michigan were lower than for A/California in both assays.

**Figure 3:**
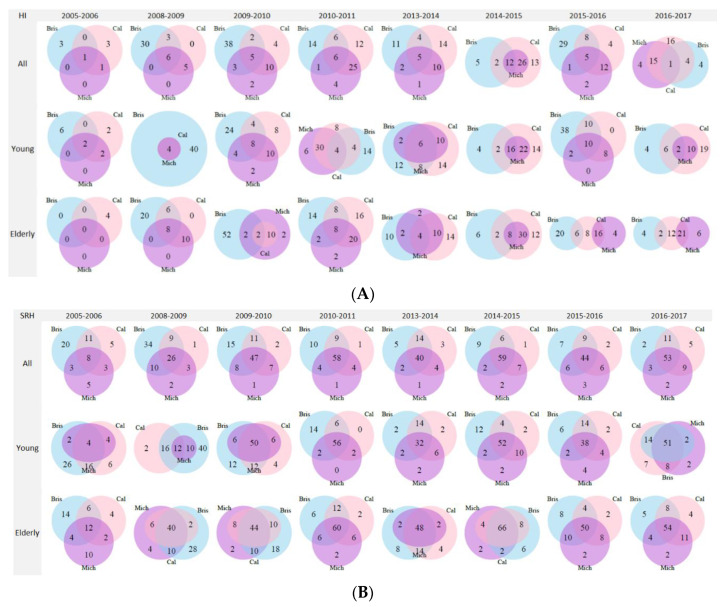
(**A**) Venn diagrams for HI proportions of subjects with protective titers (A/Brisbane, blue circle; A/California, pink circle; A/Michigan, purple circle) and combinations, by age group and season. (**B**) Venn diagrams for SRH proportions of subjects with protective titers (A/Brisbane, blue circle; A/California, pink circle; A/Michigan, purple circle) and combinations, by age group and season.

Considering the proportions of cross-protection (Figure 3A), in the 2008/2009 season HI positives were mostly positive only for A/Brisbane (30.0%, 95% CI 21.9–39.6). In the 2009/2010 season, the higher value was still represented by A/Brisbane only (38.0%, 95% CI 29.1–47.8), with a higher proportion of positives in the >65-year-old age group (52.0%, 95% CI 38.5–65.2 vs. 24%, 95% CI 14.2–37.5 in the 18–65-year-old age group, *p* = 0.004). In the same season, the second highest proportion was observed for A/California and A/Michigan (10%, 95% CI 5.3–17.6 of cross-protection), and from the 2010/2011 season onwards, the highest proportions were for A/California and A/Michigan together or for A/California only, with the exception of the 2015/2016 season.

Considering the SRH results (Figure 3B) in the 2005/2006 season, 42.0% (95% CI 33.0–52.0) of the total population already showed protective titers against A/Brisbane, 27.0% (95% CI 19.0–36.0) for A/California, and 19.0% (95% CI 13.0–28.0) for A/Michigan. The highest proportion was for positives to A/Brisbane only (20.0%, 95% CI 13.3–29.0), followed by proportion of positives for A/California and A/Brisbane (11.0%, 95% CI 6.1–18.8). Considering the >65-year-old age group, the second highest proportion was for all three strains together (12.0%, 95% CI 5.2–24.2). In the 2008/2009 season, 34.0% (95% CI 25.4–43.7) of the subjects showed positivity to A/Brisbane only. However, when considering age groups, 40% (95% CI 27.6–53.8) of >65-year-olds showed protective titers for all of the three strains. This was also reflected in the results for the total population, where 26.0% (95% CI 18.4–35.4) of subjects had protective titers against all of three strains. Starting from the 2009/2010 season onward, in both age groups, the highest proportion of subjects had protective titers against all three strains.

Overall, proportions of subjects with protective titers were significantly higher for the SRH assay across strains, seasons, and age groups than the HI assay (*p* < 0.0001) (Table 2). The proportion of SRH positives was higher in the >65-year-olds age group in comparison to the 18–65-year-old group.

A comparison between the proportion of HI and SRH positives was performed between pre- and post-outbreak seasons for each strain, with reference to the WHO vaccine strain recommendation (Table 1). Seasons 2005/2006 (pre-outbreak) and 2008/2009, and the following seasons (post-outbreak) were compared for the A/Brisbane strain, and seasons 2005/2006 and 2008/2009 (pre-outbreak) were compared to 2009/2010 and the following seasons (post-outbreak) for the A/California strain. In contrast, we found no clear definition of pre- and post-outbreak seasons for the A/Michigan strain when comparing the years before the WHO recommendation. Therefore, based on the serological results, the most reasonable season for the appearance was set as the 2014/2015 season.

**Figure 4:**
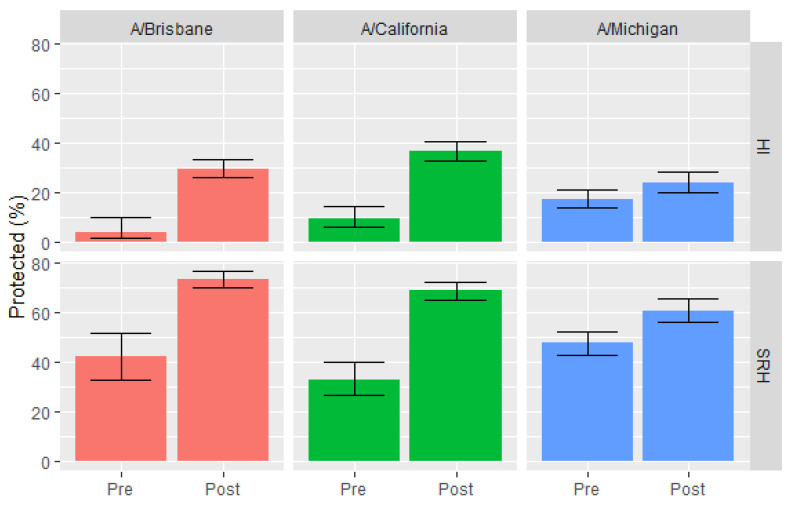
Pre- and post-outbreak seasons by strain. HI and SRH results.

HI and SRH results for A/Brisbane and A/California strains showed a clear distinction between the number of subjects with positive titers between pre- and post-outbreak seasons (*p* < 0.0001 for both assays). For the A/Michigan strain, differences between pre- and post-outbreak seasons were more evident for SRH (*p* < 0.0001) than for HI (*p* = 0.01) (Figure 4).

To provide an explanation for the observed cross-protective antibody responses, the amino acid sequences of HA were compared between the strains. The HA amino acid sequence of A/California was aligned with the HA amino acid sequences from the A/Brisbane and A/Michigan strains. The A/California strain exhibited the highest sequence identity to the A/Michigan strain (97.1% homology). In particular, the HA1 domain of A/California shared 96.0% homology with the A/Michigan strain’s HA1 domain, and 98.6% for the HA2 domain. In contrast, HA sequence identity to A/Brisbane was lower (79.8%), with 71.6% homology for HA1 and 91.8% for HA2.

## 4. Discussion

This study provided a serologically-based evaluation and interpretation of the levels of cross-reactive (and cross-protective) antibodies against H1N1 viruses circulating in the human population since 2008.

In the 2005/2006 season, low HI proportions of subjects with protective titers were observed in both age groups, as none of the H1N1 strains included in this study were circulating. The SRH results showed some protection, most probably correlating with pre-existing immunity, which was higher for A/Brisbane (42.0%) and lower for A/California and A/Michigan (27.0% and 19.0%, respectively). However, elderly adults showed higher cross-protective titers against all the three strains (12.0%) than younger adults. In the 2008/2009 season (considered pre-pandemic), the HI results clearly showed A/Brisbane as the predominant strain, especially in elderly adults (34.0%). With respect to SRH, 34.0% of the total population had protective titers against A/Brisbane; however, 40.0% of elderly adults showed cross-protective titers against all three strains, probably because of the presence of pre-existing cross-reactive antibodies against former H1N1 strains that may have contributed to protection in this age group [3]. In the 2009/2010 season, the higher proportion of protective antibodies was still A/Brisbane-specific; however, cross-protective antibodies between A/California and A/Michigan were already detectable, most likely induced by the spread of the 2009 pandemic strain. Starting from the 2009/2010 season onward, a higher proportion of SRH positive subjects showed cross-protection for all three strains. In the 2010/2011 (pandemic/post-pandemic) season and onward, higher proportions of protective antibodies were found against A/California and A/Michigan together, or A/California only, and in seasons when A/Michigan was possibly circulating.

Despite some antigenic differences, the A/California and A/Michigan strains share common epitopes. Although the WHO recommended A/Michigan as the H1N1 vaccine component in the 2017/2018 season, replacing A/California, the population already showed protection against the A/Michigan strain in the first seasons of A/California circulation. It could be concluded that there has been a co-evolution of both strains over the seasons, associated with a cross-reactivity between A/California and A/Michigan that does not allow a discrimination of the exact season when A/Michigan strain became predominant. The data described here indicate that antibodies raised against A/California are cross-protective against A/Michigan. As previously observed [22,23], the antigenic drift of HA of the 2009 pandemic H1N1 strain was not observed with ferret antisera, although more recent 2009 pandemic H1N1-like strains, such as A/Michigan, are antigenically different to the vaccine strain (A/California).

In elderly adults, protective antibody titers against A/Brisbane strain were associated with cross-protective antibodies against A/Michigan and A/California strains as well. One possible explanation is that, despite the HA from the pandemic H1N1 virus not being antigenically similar to any previous human seasonal influenza virus [3], the adaptation to human hosts led to common conserved epitopes, which can be recognized by the broader antibody repertoire of elderly adults and, as such, result in higher titers in this age group. This is also supported by the observation that although the elderly may show lower titers than younger adults against homologous strains, they showed higher cross-reactions (and cross-protective titers) against heterologous strains (drifted strains). These findings were supported by other studies [9,11], which have shown that immunological priming by previous exposure to influenza strains participates in the immune response to an antigenically-related strain and increases with age.

The analysis performed in this study supports the use of the two different assays, HI and SRH, in parallel, as it allows investigations into the antigenic nature of three different virus types in two distinct populations, younger and elderly adults, in an unusual scenario; (i) a seasonal influenza H1N1 strain of human origin as a representative of classical drifted A/H1N1 strains, A/Brisbane; (ii) an influenza H1N1 virus of animal origin (swine) that caused a pandemic in humans in 2009/2010 and then became a classical seasonal human influenza virus replacing the former seasonal human H1N1 strain, A/California; and (iii) a drift variant of the original pandemic strain due to antigenic changes resulting from frequent circulation in the human population over several seasons, A/Michigan. This study showed that the HI assay is an optimal assay for determination and detection of new seasonal drift strains, as shown in the pre/post-outbreak A/Brisbane and pre/post-outbreak A/California seasons. Based on its specificity, the HI assay is able to discriminate which new strain is circulating following vaccine recommendation. In accordance with the HI results of this study in both age groups, it is possible to recognize peaks of prevalence through the seasons that, in the majority of cases, are in accordance with virologic surveillance data. SRH immunity seems to accumulate over the seasons, with minor variations in the proportion of positive subjects. Antibodies against viral epitopes, recognized by SRH, are more stable between seasons and strains, as the SRH assay is able to detect antibodies directed against potentially more conserved epitopes between different strains, such as antibodies against HA2 [15], allowing the detection of a broader range of functional antibodies that contribute to previous immunity. This previous immunity was observed to be more pronounced in the elderly, as already highlighted by previous studies [3,7,8,9,10,11]. The SRH assay, in addition, is an excellent tool for determining the presence of pre-existing immunity against drifted and actual circulating seasonal strains. This would allow a potential prediction of booster ability against newly emerging drifted strains.

## Figures and Tables

**Table 1:** Influenza A/H1N1 strain egg-based vaccine composition Northern Hemisphere (NH) for the 2005/2006–2020/2021 seasons. Shown in bold the first time the strains considered in this study were included in WHO vaccine recommendation.

NH Season	A/H1N1 Strain
2005/2006	A/New Caledonia/20/99-like
2006/2007	A/New Caledonia/20/99-like
2007/2008	A/Solomon Island/3/2005-like
2008/2009	**A/Brisbane/59/2007-like**
2009/2010	A/Brisbane/59/2007-like
2010/2011	**A/California/7/2009-like**
2011/2012	A/California/7/2009-like
2012/2013	A/California/7/2009-like
2013/2014	A/California/7/2009-like
2014/2015	A/California/7/2009-like
2015/2016	A/California/7/2009-like
2016/2017	A/California/7/2009-like
2017/2018	**A/Michigan/45/2015-like**
2018/2019	A/Michigan/45/2015-like
2019/2020	A/Brisbane/02/2018-like
2020/2021	A/Guangdong-Maonan/SWL1536/2019-like

**Table 2:** Proportions of positives (%) by strain, age group, and assay.

**Age Group**	**Assay**	**A/Brisbane**	**A/California**	**A/Michigan**
18–65	HI	29.50	30.00	20.25
>65	HI	23.25	29.75	20.75
18–65	SRH	66.17	58.95	46.91
>65	SRH	72.17	63.19	61.43
